# Childhood adversity and psychosis: generalised or specific effects?

**DOI:** 10.1017/S204579601500044X

**Published:** 2015-07-09

**Authors:** E. Longden, M. Sampson, J. Read

**Affiliations:** 1 Institute of Psychology, Health and Society, University of Liverpool, Liverpool, UK; 2 Early Intervention Services, Auckland District Health Board, Auckland, New Zealand; 3 Department of Psychological Sciences, Swinburne University of Technology, Melbourne, Australia

**Keywords:** Childhood abuse, maltreatment, schizophrenia, specificity

## Abstract

**Background.:**

This study examines relationships between childhood adversity and the presence of characteristic symptoms of schizophrenia. It was hypothesised that total adversity exposures would be significantly higher in individuals exhibiting these symptoms relative to patients without. Recent proposals that differential associations exist between specific psychotic symptoms and specific adversities was also tested, namely: sexual abuse and hallucinations, physical abuse and delusions, and fostering/adoption and delusions.

**Method.:**

Data were collected through auditing 251 randomly selected medical records, drawn from adult patients in New Zealand community mental health centres. Information was extracted on presence and subtype of psychotic symptoms and exposure to ten types of childhood adversity, including five types of abuse and neglect.

**Results.:**

Adversity exposure was significantly higher in patients experiencing hallucinations in general, voice hearing, command hallucinations, visions, delusions in general, paranoid delusions and negative symptoms than in patients without these symptoms. There was no difference in adversity exposure in patients with and without tactile/olfactory hallucinations, grandiose delusions or thought disorder. Indication of a dose–response relationship was detected, in that total number of adversities significantly predicted total number of psychotic symptoms. Although fostering/adoption was associated with paranoid delusions, the hypothesised specificity between sexual abuse and hallucinations, and physical abuse and delusions, was not found. The two adversities showing the largest number of associations with psychotic symptoms were poverty and being fostered/adopted.

**Conclusions.:**

The current data are consistent with a model of global and cumulative adversity, in which multiple exposures may intensify psychosis risk beyond the impact of single events. Implications for clinical intervention are discussed.

Exposure to childhood loss, stress and victimisation has been extensively studied as a risk factor for psychosis, with meta-analyses reporting odds ratios of between 2.78 (Varese *et al*. 2012) and 3.60 (Matheson *et al*. 2013) for multiple forms of adversity. This relationship has been replicated across different populations and study designs, with further confirmation emanating from consistent findings of a dose–response; that is, an increase in risk according to number or severity of exposures. For example, analysis of two large community samples found evidence of a dose–response effect for cumulative trauma exposure (childhood neglect, physical abuse, physical attack or assault, rape, sexual molestation) and psychosis likelihood (Shevlin *et al*. 2008); while a more recent population-based household survey reported that childhood physical or sexual abuse plus incidence of other adverse life events (e.g., bereavement, serious accident or injury, witnessing violence) combine ‘synergistically’ to increase psychosis risk beyond the effect of each adversity individually (Morgan *et al*. 2014).

Several propositions have been made for the mechanisms by which cumulative adversity confers psychosis risk, including the suggestion that exposures create vulnerability to psychotic experience through toxic effects on biological (Read *et al*. 2014), cognitive (Gracie *et al*. 2007) and affective (Fisher *et al*. 2013) systems, which in turn may be amplified by additive environmental stressors over time. In this respect, while research has generally prioritised factors such as childhood sexual abuse (CSA), physical abuse (CPA) and physical neglect (CPN), greater attention is now being paid to other forms of adversity. For example, recent meta-analyses have proposed a heightened risk of psychotic symptoms in association with such factors as peer bullying (van Dam *et al*. 2012), parental communication deviance (de Sousa *et al*. 2014) and urbanicity (Vassos *et al*. 2012). Empirical work with large cross-sectional datasets have likewise emphasised the potential role of deprivation and social inequalities (Wickham *et al*. 2014) and being raised in institutional care (Bentall *et al*. 2012) in predicting psychotic symptoms, as well as the role of attachment quality in mediating between adversity exposure and subsequent psychosis (Sitko *et al*. 2014).

In addition to aggregate adversity and disadvantage, research has also examined whether a degree of specificity may exist between particular types of childhood adversity and particular symptoms of psychosis. For example, analysis of the UK 2007 Adult Psychiatric Morbidity Survey found that childhood rape was associated with an increased risk of hallucinations (but not paranoid delusions) when controlling for other adversities and psychotic symptoms; whereas paranoia was specifically associated with CPA and being raised in institutional care when adjusting for adversity exposure and co-occurring hallucinations (Bentall *et al*. 2012). Analysis of the US National Comorbidity Survey (Sitko *et al*. 2014) likewise reported differential associations between hallucinations and CSA, and paranoid beliefs and neglect (conceived by the authors as comparable with institutional care, in that a failure to adequately meet the child's emotional, physical or intellectual needs is indicative of attachment disruption and can induce the same severe impact as physical separation). Another study (Shevlin *et al*. 2014), which analysed data from 3142 UK prison inmates, likewise found that CSA produced the highest odds ratio for hallucinations (2.37) with paranoia specifically predicted by childhood bullying (1.99) and being raised in institutional care (1.49). The associations remained stable when controlling for prison-based adversity exposure, suggesting that they are not confounded by the experience of substantial adulthood adversity. These investigations are notable for their large samples and statistical adjustment for potential confounders. However, less controlled observational research has also found that CSA survivors may be more likely to report hallucinations, particularly auditory, relative to delusions (Read & Argyle, 1999; Hainsworth *et al*. 2011; Sheffield *et al*. 2013).

Taken together, this literature reflects a wider conceptual shift in psychosis research that advocates ‘complaint-orientated’ (Bentall, 2006) or ‘staging and profiling’ (Wigman *et al*. 2013) approaches. That is, that phenomenon such as hallucinations and delusions can be independently examined in their own right, rather than subsuming their study within the context of diagnostic categories such as ‘schizophrenia,’ which is a heterogeneous, disjunctive construct with poor reliability (Read, 2013*a*). Refining accounts of specific associations between adversity exposures and outcomes is an important endeavour in terms of promoting better understandings of how particular risk factors might impact on different biological and psychological mechanisms to create a psychosis pathway. For example, Bentall & Fernyhough (2008) posit that paranoia is connected with heightened threat expectancy and a propensity to attribute adverse events to external sources; which are psychological mechanisms suggested to logically result from disempowerment (e.g., CPA) and disrupted attachment relationships (e.g., institutional care). In terms of voice hearing, these authors also suggest that formative adversity, particularly CSA, may hinder the source-monitoring mechanisms required to differentiate between external and self-generated stimuli, possibly in combination with adversity-induced dissociation. Although precise adversity-related and symptom-specific accounts of psychosis are still provisional, improved knowledge of these mechanisms could offer substantial clinical implications in terms of tailoring both pharmacological (e.g., ‘rational drug design’ targeted at component symptoms of psychosis: Fibiger, 2012) and psychosocial (e.g., addressing specific processes such as dissociation or threat salience: Bentall *et al*. 2014).

## Study aims

The aim of the current study was to examine relationships between a broad range of childhood adversities (including the usual five main types of abuse and neglect, but also the less commonly researched variable of fostering/adoption) and the presence of DSM-IV characteristic symptoms of schizophrenia: hallucinations, delusions, thought disorder, negative symptoms and catatonia. It was hypothesised that the number of childhood adversity exposures would be significantly higher in individuals exhibiting these symptoms relative to patients who did not display signs of psychosis. In addition, it was hypothesised that specific associations would be identified between CSA and hallucinations; and between CPA, fostering/adoption (as an attachment disruption) and delusions.

## Method

### Procedure

Data were collected through reviewing electronic medical records of 251 adult service-users drawn from four urban community mental health centres (CMHCs) in New Zealand. Records were randomly selected from a computer-generated pool of 850 potential files. Files reporting no face-to-face contact with staff, or only face-to-face contact in a crisis scenario (e.g., a police station) or with non-CMHC staff were excluded. Files active for less than 3 days were also excluded on the grounds that a full assessment with a healthcare worker was unlikely to have taken place. A total of 141 files were omitted based on these criteria. All retained files were read in their entirety. Data were extracted by a Clinical Psychology graduate trainee (MS) and a registered clinical psychologist (JR) and documented on a specifically designed recording instrument that included: demographic characteristics; primary DSM-IV diagnosis; subtype and content of characteristic symptoms of DSM-IV criteria schizophrenia; and exposure to 13 different types of childhood adversity (CSA, CPA, CPN, emotional abuse [CEA], emotional neglect [CEN], bullying, poverty, fostering/adoption, death of a parent/caregiver, witnessing domestic violence, mental illness in a parent/caregiver, alcohol or substance use of a parent/caregiver, divorce of parents/caregivers). Childhood adversity was classified as that occurring prior to age 18.

### Reliability

Owing to the observational nature of the data, operational definitions of the types of adversity examined were primarily based on that identified by clinicians and clients. For example, records stating ‘sexually abused as a child’ were considered sufficient to code for abuse having occurred. Files in which life history sections had not been completed were noted as ‘no abuse history taken’ and marked as missing data. If information suggestive of adversity was considered inconclusive, files were independently inspected by two researchers (MS and JR). To be included in the analysis, cases had to be rated as ‘95% or more probable’ to have occurred by both raters. Thirty-two files were judged to contain ambiguous information, in which agreement was reached in 29 cases (inter-rater reliability 91%, *κ* = 0.81). Examples of cases in which abuse was rated as <95% likely to have occurred included the statement ‘*violent and abusive father*,’ on the grounds that the description was vague and did not ascertain whether the father was abusive towards the client specifically, and ‘*reported traumatic childhood*’ with no clarifying details. An example of cases rated as 95% or more likely to have occurred stated ‘*father began to drink heavily and took up the use of severe and frequent corporal punishment*’ and ‘*made to watch sexual activities as a child*.’ In total, adversities in 14 of the 32 ambiguous files were rated as highly likely by both researchers and retained in the analysis.

The same criterion of 95% certainty estimation was adopted for coding psychotic symptoms. Information in 13 files was considered ambiguous, for which independent inter-rater agreement (EL and JR) was 100%. Examples of excluded data included ‘*mildly grandiose – entitlement and her being ‘special’*’ and ‘*talks about conspiracy theories*’ (for delusions), ‘*racing thoughts that are difficult to stop*’ and ‘*rambling thoughts*’ (for thought disorder) and ‘*talk*[*ed*] *of often hearing people climbing over the fence at home but when she looks no one is there*’ (for hallucinations). All 13 of these ambiguous cases were excluded from the analysis.

Data from the recording forms were manually entered into SPSS v.20.0 (IBM Corp., 2011) for analysis. To minimise data entry errors, 50 files (20.0%) were selected at random and double-checked against the hard-copy data collection from. Data entry agreement was 99.7%.

### Statistical analysis

Between-group differences in clinical presentation and adversity exposure were assessed using Mann–Whitney *U*-tests to correct for the unequal group sizes and irregular data distributions. However, parametric statistics were used for descriptive summaries as they have closer correspondence with real world values than their non-parametric counterparts. Corresponding effect sizes were calculated using Cohen's *r.* Associations between specific clinical presentations and specific adversities were analysed using the phi-coefficient and unadjusted odds ratios. Associations between total adversity exposures and comorbidity for psychotic symptoms were assessed using bivariate linear regression.

Three of the 13 childhood adversity variables had more than 50% missing values and were not retained (bullying, witnessing domestic violence and alcohol or substance use of a parent/caregiver), leaving ten types of events for analysis: CSA, CPA, CPN, CEA, CEN, poverty, fostering/adoption, death of a parent/caregiver, mental illness of a parent/caregiver and divorce of parents/caregivers. Because of the large number of planned comparisons, alpha was set at a more stringent *p* ≤ 0.025 level to reduce the likelihood of type 1 (false positive) error.

## Results

### Participants

Demographic and clinical features of the sample are presented in Table 1. Participants consisted of 122 women and 129 men, with a mean age of 35.7 years (s.d.: 12.36). The majority were either New Zealand European (55.3%) or Māori (23.9%), and were commonly single (48.6%) and either unemployed or in receipt of sickness benefits (52.2%). The most frequent diagnoses were either mood (45.4%) or psychotic (23.1%) disorders.

**Table 1.** Demographic and clinical characteristics of the sample

Total *n* = 251	*n*	%	
Gender			
Female	122	48.6	
Male	129	51.4	
Ethnicity			
New Zealand European	139	55.3	
Māori	60	23.9	
Pacific Islander	22	8.8	
Other	27	10.8	
Data missing	3	1.2	
Marital status			
Single	122	48.6	
Married	45	17.9	
Co-habiting/*de facto* relationship	39	15.5	
Divorced/separated	36	14.3	
Widowed	3	1.2	
Data missing	6	2.4	
Level of education			
No formal qualifications	65	25.9	
University degree or diploma	47	18.7	
University entrance	44	17.5	
School certificate	31	12.4	
Trade certificate	9	3.6	
Data missing	55	21.9	
Employment status			
Unemployed/sickness benefits	131	52.2	
Full-time employed	70	27.9	
Student	19	7.6	
Part-time employed	18	7.2	
Self-employed	8	3.2	
Maternity leave	1	0.4	
Data missing	4	1.6	
Primary diagnosis			
Mood disorder	114	45.4	
Psychotic disorder	58	23.1	
Anxiety disorder	19	7.6	
Other	35	13.9	
No diagnosis	25	9.9	
	Mean	s.d.	Range
Age	35.66	12.36	18–63

### Prevalence and characteristics of childhood adversities

Of the examined files, 141 (56.2%) reported at least one form of childhood abuse or neglect, the most common of which was CPA (89; 35.5%), followed by CEA (88; 35.1%), CSA (82; 32.7%), CEN (53; 21.1%) and CPN (18; 7.2%). At least one of the remaining five adversities were documented in 175 (69.7%) files, of which mental illness in a parent/caregiver was most prevalent (102; 40.6%) followed by divorce of parents/caregivers (100; 39.8%); fostering/adoption (38; 15.1%); poverty (31; 12.4%) and death of a parent/caregiver (28; 11.2%).

### Prevalence and characteristics of psychotic symptoms

The mean number of psychotic symptoms reported across the sample was 3.47 (s.d.: 1.46). Of the charts examined, at least one form of hallucination was noted in 119 cases (47.4%). Auditory hallucinations were the most common modality (114; 95.8%: 45.4% of whole sample), of which 38 (31.9%; 15.1% of whole sample) were command hallucinations. Visions (48; 40.3%: 19.1% of whole sample) and tactile/olfactory hallucinations (15; 12.6%: 6.0% of whole sample) were less commonly reported. At least one type of delusion was noted in 110 cases (43.8%), which were more likely to be paranoid (104; 94.5%: 41.4% of whole sample) than grandiose (40; 36.4%: 15.9% of whole sample). Thought disorder was recorded in 59 cases (23.5%) followed by 47 instances of negative symptoms (18.7%). Catatonic symptoms were noted in only nine cases (3.6%), and were therefore not included in inferential analyses.

### Group differences in adversity exposure

The total number of adverse childhood events was compared for each psychotic symptom and subtype, using patients without these symptoms as the reference group. The number of adversities was significantly higher in patients reporting hallucinations in general, voice hearing, visions, command hallucinations, delusions in general, paranoid delusions and negative symptoms (see Table 2). Using Cohen's *r* criteria (1988), wherein ≥0.5 is considered a large effect, these differences were associated with moderate to small effect sizes. There were no significant differences in childhood adversity exposure in patients with and without thought disorder, tactile/olfactory hallucinations or grandiose delusions.

**Table 2.** Group differences and associated effect sizes in mean number of childhood adversity exposures in participants with and without psychotic symptoms

Clinical characteristics	Childhood adversity exposures	*U*-statistic	*r*
	M (s.d.)		
Any hallucination			
Yes = 88	3.91 (2.00)		
No = 73	2.74 (1.91)	2107.00***	0.30
Voices			
Yes (*n* = 84)	3.87 (1.99)		
No (*n* = 77)	2.81 (1.99)	2242.00***	0.27
Command hallucinations			
Yes (*n* = 32)	3.94 (2.05)		
No (*n* = 102)	3.07 (2.02)	1208.50*	0.20
Visions			
Yes (*n* = 36)	4.08 (2.14)		
No (*n* = 125)	3.15 (1.98)	1701.00*	0.18
Tactile/olfactory			
Yes (*n* = 12)	3.50 (1.88)		
No (*n* = 149)	3.35 (2.07)	806.00	
Any delusion			
Yes (*n* = 73)	3.88 (2.05)		
No (*n* = 88)	2.93 (1.97)	2384.50**	0.22
Paranoid			
Yes (*n* = 69)	3.90 (2.03)		
No (*n* = 92)	2.96 (1.99)	2346.00**	0.23
Grandiose			
Yes (*n* = 20)	4.25 (2.05)		
No (*n* = 141)	3.23 (2.03)	1037.50	
Thought disorder			
Yes (*n* = 38)	3.74 (1.78)		
No (*n* = 123)	3.24 (2.12)	2001.50	
Negative symptoms			
Yes (*n* = 28)	4.49 (1.81)		
No (*n* = 133)	3.14 (2.04)	1169.00**	0.25

Significance level: *p ≤ 0.025; **p < 0.005; ***p < 0.001.

To determine whether combined adversity exposures increased the likelihood of higher comorbidity, a bivariate linear regression was conducted using total number of psychotic symptoms as the criterion variable and total number of adversities as the predictor. The equation was significant (*F*(1,159) = 14.92, *p* = 0.001) and indicated that patients with a higher number of adversity exposures reported a greater amount of psychotic symptoms (regression equation: 1.05 + 0.28 (× total adversities); *R*^2^ = 0.09).

### Associations between specific adversities and specific psychotic symptoms

Table 3 presents associations and respective odds ratios and confidence intervals between psychotic symptoms and adversity exposures. Contrary to predictions, there was no significant association between CSA and any hallucination subtype, or CPA and either type of delusion. However, the hypothesis that exposure to fostering/adoption would increase the likelihood of experiencing paranoid delusions was supported. The association between the latter and grandiose delusions also approached significance, but did not meet the adjusted alpha level set for this study (*p* = 0.028).

**Table 3.** Phi-coefficients (φ) and significant odds ratios between specific psychotic symptoms and specific childhood adversities

	CSA	CPA		CEA		CEN	CPN	F/A		P		PD	D	MI
	*φ*	*φ*	Odds ratio (95% CI)	*φ*	Odds ratio (95% CI)	*φ*	*φ*	*φ*	Odds ratio (95% CI)	*φ*	Odds ratio (95% CI)	*φ*	*φ*	*φ*
Any hallucination	NS	0.18*	2.11 (1.10–4.07)	NS		NS	NS	0.23***	3.64 (1.66–7.95)	0.24**	3.41 (1.45–8.06)	NS	NS	NS
Voices	NS	NS		NS		NS	NS	0.20**	2.93 (1.39–6.20)	0.25**	3.67 (1.55–8.67)	NS	NS	NS
Command hallucinations	NS	0.23*	3.35 (1.32–8.50)	NS		NS	NS	0.20*	3.21 (1.35–7.65)	0.29**	4.56 (1.68–12.40)	NS	NS	NS
Visions	NS	NS		NS		NS	NS	NS		NS		NS	NS	NS
Tactile/olfactory	NS	NS		NS		NS	NS	NS		NS		NS	NS	NS
Any delusion	NS	NS		0.18*	2.11 (1.09–4.07)	NS	NS	0.25***	4.16 (1.90–9.12)	NS		NS	NS	NS
Paranoid	NS	NS		0.19*	2.20 (1.13–4.26)	NS	NS	0.24***	3.61 (1.70–7.65)	0.19*	2.56 (1.13–5.77)	NS	NS	NS
Grandiose	NS	NS		NS		NS	NS	NS		NS		NS	NS	NS
Thought disorder	NS	NS		NS		NS	NS	NS		NS		NS	NS	NS
Negative symptoms	NS	0.27***	5.63 (1.84–17.16)	0.22*	3.94 (1.40–11.11)	NS	NS	0.20**	3.16 (1.43–6.93)	0.21*	3.21 (1.27–8.11)	NS	NS	NS

CSA, childhood sexual abuse; CPA, childhood physical abuse; CEA, childhood emotional abuse; CEN, childhood emotional neglect; CPN, childhood physical neglect; F/A, fostering/adoption; P, poverty; PD, death of a parent/caregiver; D, divorce of parents/caregivers; MI, mental illness in a parent/caregiver; NS, not significant.

Significance level: *p ≤ 0.025; ** *p* *<* 0.005; ***p < 0.001.

CSA, CEN, CPN, death of a parent/caregiver, divorce of parents/caregivers and mental illness in a parent/caregiver were not specifically associated with any psychotic symptoms. CPA was associated with an increased probability of reporting hallucinations in general, command hallucinations and negative symptoms; and CEA was associated with delusions, in general, paranoid delusions and negative symptoms.

The two adversities with the largest number of significant associations were poverty and fostering/adoption, which both showed the same specific relationships with hallucinations in general, voice hearing, command hallucinations, paranoid delusions and negative symptoms.

## Discussion

The data confirm existing evidence that increased childhood adversity exposure is related to more severe psychiatric outcomes in adulthood, including psychosis (e.g., Varese *et al*. 2012), and that this association follows a dose-dependent pattern (e.g., Shevlin *et al*. 2008). However, although fostering/adoption was significantly associated with paranoid delusions, no relationship was found between CSA and any type of hallucination, or CPA and paranoid delusions. Thus the hypothesis (e.g., Bentall *et al*. 2012; Shevlin *et al*. 2014; Sitko *et al*. 2014) that differential associations exist between these particular adversities and psychotic symptoms was only partially supported.

Contrary to the specificity model, the current data are more consistent with a model of global cumulative adversity, in that number of exposures was significantly higher in patients experiencing hallucinations in general, voice hearing, command hallucinations, visions, delusions in general, paranoid delusions and negative symptoms (although not tactile/olfactory hallucinations, grandiose delusions or thought disorder) than patients without these symptoms; and that greater adversity exposure was associated with greater comorbidity.

The suggestion that combinations of different adversities might intensify psychosis risk beyond the impact of individual stressors has recently been examined in two large epidemiological studies. The first, a population-based survey of 1680 individuals in the UK, reported strong evidence for cumulative, ‘synergistic’ effects of abuse and adversity that were associated with a two- to fourfold increase of reporting low-level psychotic experience in the year preceding assessment (Morgan *et al*. 2014). The second, a combined sample for the Dutch NEMESIS studies (*n* = 13 722), likewise found strong, significant associations between childhood adversity and hallucinations, delusions, voice hearing and paranoia (van Nierop *et al*. 2014). However, when specific associations between CPA, CSA and foster care were examined using mixed-effects regression (capable of determining specific associations amongst multiple correlated outcomes), no differential relationships for delusions and hallucinations were found. Although both these studies were conducted within the general population, our random sample of psychiatric service-users yields a broadly comparable pattern of results in that more significant associations were found for multiple adversity exposure rather than specific associations between particular events and particular clinical outcomes.

It may therefore be notable that the two adversities in the current analysis with the largest number of significant associations with psychotic symptoms – poverty and fostering/adoption – could be seen as proxies for a range of more general (and cumulative) environmental risks and disadvantages (Neil, 2000; Read, 2010; Read *et al*. 2013). For example, requiring substitute parental care is indicative of dysfunction, loss or stress in the family of origin and is associated with poorer long-term outcomes for adult adjustment, wellbeing and self-sufficiency (Buehler *et al*. 2000). This result therefore lends further support to a growing body of literature indicating that attachment quality and disruptions in attachment behaviours (particularly that occurring in early childhood) can contribute to the development of adulthood psychosis (Read & Gumley, 2008; Harder, 2014; Sitko *et al*. 2014).

Poverty has been shown to be a strong predictor of psychosis for more than 60 years (Read, 2010; Read *et al*. 2013). Like fostering/adoption, it is also strongly associated with a greater incidence of childhood maltreatment (Drake & Pandey, 1996; Gillham *et al*. 1998; Lee & Goerge, 1999) and chronic stress dysregulation (Evans & Kim, 2007). Complex interactions have also been proposed between inequality, deprivation, stress, discrimination, mistrust and lack of social support, as predictors of affective and non-affective psychosis (Wickham *et al*. 2014). Although the cross-sectional and correlational nature of the current data prohibits any causal assumptions, the consistency between these two variables is striking in that both exhibited the same pattern of significant associations with hallucinations in general, voice hearing, command hallucinations, paranoid delusions and negative symptoms.

In contrast, we found few significant associations between any psychotic symptom and the five types of abuse and neglect: none for CSA, CEN, or CPN and only three for CPA and CEA. Given the substantial literature associating these experiences with psychosis (see Read, 2013*b*), this appears to be an unexpected result. However, it perhaps becomes more explicable when considering that formative exposure to any of these events can create vulnerability and stress sensitisation (Read *et al*. 2014) that may augment psychosis risk over time through subsequent social stressors and hardships. For example, recent large-scale epidemiological studies have found that factors like social marginalisation (Boyda *et al*. 2014) and attachment quality (Sitko *et al*. 2014) mediate associations between interpersonal adversity and psychotic symptoms. As noted by Morgan *et al*. (2014) ‘In so far as adverse social experiences tend to cluster in individuals, families and neighbourhoods and persist over time, it is essential to move on from identifying specific social and environmental risk factors for psychosis to examine the impact of multiple exposures, how they interact and the mechanisms through which they exert their effects’ (p. 352). In this respect research that incorporates a broader spectrum of stressors beyond abuse and neglect into their analyses is an important endeavour.

### Clinical implications

Despite guidelines emphasising the need to routinely assess adversity exposure in psychiatric service-users (National Health Service Confederation, 2008), research suggests such recommendations are often not implemented (Read *et al*. 2007; Fisher *et al*. 2011; Hepworth & McGowan, 2013).While staff should not pre-suppose a history of maltreatment unless confirmed by the client, the current findings support the contention that clinicians should receive support and training for making routine evaluations for possible experiences of maltreatment. This is particularly important given the significant under-detection of posttraumatic stress in patients diagnosed with psychosis (Salyers *et al*. 2004; Lommen & Restifo, 2009; Mauritz *et al*. 2013), and that such individuals are less likely to receive an appropriate clinical response (e.g., trauma-focused interventions) relative to those with non-psychotic diagnoses (Agar & Read, 2002; Salyers *et al*. 2004; Grubaugh *et al*. 2011) especially in instances where healthcare workers have strong convictions about biogenetic aetiology (Read & Fraser, 1998; Young *et al*. 2001; Agar & Read, 2002).

The feasibility and utility of non-pharmacological approaches to psychosis have only become an area of systematic research interest within the last few decades and, with the exception of CBT, robust evidence for their efficacy (e.g., Cochrane reviews) is limited. There is a clear need for well-defined therapeutic approaches that can address the sequalae of interpersonal adversity in patients diagnosed with psychosis, particularly given the frequency with which previous victimisation may manifest in the content and maintenance of positive symptoms (Hardy *et al*. 2005; Raune *et al*. 2006; Thompson *et al*. 2010; Falukozi & Addington, 2012; Corstens & Longden, 2013). One promising approach (Read *et al*. 2003) is combining trauma-focused therapeutic models (e.g., Herman, 1992; Ross & Halpern, 2009; Bacon & Kennedy, 2014) with treatments that have established effectiveness in alleviating psychotic symptoms (e.g., acceptance and commitment therapy (Gaudiano & Herbert, 2006), cognitive therapy (Morrison *et al*. 2014), compassion-focused therapy (Braehler *et al*. 2013), early intervention strategies (McGorry *et al*. 2008) and Open Dialogue (Seikkula *et al*. 2011)).

Despite the attractive parsimony of unique associations between particular symptoms and stressors, a model of cumulative adversity also reiterates the inherent difficulty of devising causal pathways from specific events to specific clinical outcomes. It is important that therapeutic approaches can accommodate such heterogeneity, namely by acknowledging the complex, often idiosyncratic, processes that result in psychosis, while also recognising the limitations of trying to create predictable pathways from psychosocial events to subjective manifestations of distress. Individualised interventions, such as psychological formulation (e.g., Johnstone & Dallos, 2006; British Psychological Society Division of Clinical Psychology, 2011; Longden *et al*. 2012), represent one such ‘bottom-up’ strategy that can tailor therapeutic responses to the unique combination of social and emotional conflicts experienced by a particular client.

### Limitations

The current findings must be interpreted in view of the study limitations, most notably the nature of the data. Medical record auditing is ultimately reliant on what is documented by healthcare workers, and while adversity prevalence in the current study is comparable with that of existing reviews (Wurr & Partridge, 1996; Read, 1998; Read *et al*. 2003) it is likely that some cases were unidentified. This means that both patients with and without psychotic symptoms may have experienced higher adversity rates than are currently reported. This may particularly be the case for neglect which can be identified less frequently by care services, including psychiatric facilities (Horwath, 2007). For example, Rossiter *et al*. (2015) have reported that when comparing the content of clinical records with structured measurement (the Childhood Trauma Questionnaire (CTQ): Bernstein & Fink, 1998) emotional and physical neglect were, respectively, 4.7 and 8.9 times more likely to be detected using the CTQ.

Aside from the assumption that adversity had been disclosed, believed, and recorded by the assessing clinician, the research had no formal contingencies for assessing the validity of reported exposures on a case-by-case basis. Accounts of childhood adversity may be vulnerable to contamination from factors such as traumatic amnesia (Freyd, 1994) and re-interpretive biases (Lewinsohn & Rosenbaum, 1987) in adulthood, which in psychosis may be further complicated by processes such as cognitive impairments and impaired reality-testing. Nevertheless, it should be noted that retrospective accounts of adversity among groups with complex mental health problems have proven sufficiently valid and reliable to justify the use of such data (Herman & Schatzow, 1987; Dill *et al*. 1991; Meyer *et al*. 1996; Goodman *et al*. 1999; Fisher *et al*. 2011) with one study reporting that erroneous reports of sexual victimisation are no different between patients diagnosed with schizophrenia and the general population (Darves-Bornoz *et al*. 1995).

Classification of psychotic symptoms was likewise reliant on the assessment of healthcare workers, and therefore did not reflect judgements trained to standardised levels of reliability for DSM-IV criteria. However, while independent and blinded diagnostic assessment would have been preferable, the current results do retain ecological validity in that they reflect how symptoms are assessed and classified in real-world clinical practice.

Finally, the relatively small sample, as well as numerous missing data values, meant we were not able to control for co-occurring psychotic symptoms as part of the specificity analysis. In addition, the largely categorical data prohibited the use of more rigorous inferential statistical models. It should also be noted that the large number of analyses increased the probability of type one errors, although this was addressed by avoiding *post hoc* testing and adjusting alpha to a more stringent level.
